# Editorial: Pharmacological advances to treat pathological pain

**DOI:** 10.3389/fphar.2025.1571059

**Published:** 2025-02-18

**Authors:** Célia Duarte Cruz, Sara Marchesan Oliveira, Indiara Brusco

**Affiliations:** ^1^ Department of Biomedicine, Experimental Biology Unit, Faculty of Medicine of Porto, University of Porto, Porto, Portugal; ^2^ Pain Neurobiology, Instituto de Investigação e Inovação em Saúde‐i3S and IBMC, Universidade do Porto, Porto, Portugal; ^3^ Graduate Program in Biological Sciences: Toxicological Biochemistry, Centre of Natural and Exact Sciences, Federal University of Santa Maria, Santa Maria, Rio Grande do Sul, Brazil; ^4^ Graduate Program in Environmental Sciences, Community University of Chapecó Region, Chapecó, Santa Catarina, Brazil

**Keywords:** neuropathic pain, inflammatory pain, postoperative pain, pain mechanisms, new analgesics

The International Association for the Study of Pain classifies pain as nociceptive, neuropathic, and nociplastic (Raja et al., 2020), while some researchers also include inflammatory pain (Costigan et al., 2009; Woolf, 2020; Jayakar et al., 2021). Nociceptive pain is a physiological event driven by the activation of high-threshold primary sensory neurons. It occurs in response to potentially noxious stimuli, including intense heat or cold, chemical irritants, and excessive mechanical force (Costigan et al., 2009; Jayakar et al., 2021). Inflammatory pain results from tissue injury and the subsequent inflammatory response, where inflammatory mediators activate/sensitize nociceptors (Woolf, 2020; Jayakar et al., 2021). Acute inflammatory pain is adaptive and helps to deal with the consequences of damage and protect the injured tissue, but it needs to be reduced to a manageable level, for example, after surgeries, and to prevent pain chronification (Costigan et al., 2009; Jayakar et al., 2021). Typically, inflammatory pain disappears after injury resolution, but in chronic inflammatory conditions, such as rheumatoid arthritis, the ongoing pain is maladaptive and pathologic and constitutes a clinical problem *per se* (Jayakar et al., 2021).

In contrast to nociceptive and inflammatory pains, neuropathic and nociplastic pains are always pathological. Pain is not a symptom but is a disease state itself (Jayakar et al., 2021). Neuropathic pain results from injury or disease of the somatosensory nervous system, such as nerve trauma, diabetes- or chemotherapy-induced neuropathy, spinal cord injury, or stroke (Costigan et al., 2009; Woolf, 2020). Nociplastic pain, on the other hand, occurs in the absence of noxious stimuli, active inflammation, or detectable damage to the nervous system (e.g., tension-type headache, irritable bowel syndrome, fibromyalgia). The mechanisms underlying nociplastic pain are not entirely understood, but there is altered functioning of the nervous system and pain modulation (Fitzcharles et al., 2021; Jayakar et al., 2021). Management of neuropathic and/or nociplastic pain is challenging, and an improved understanding of the mechanisms is essential to developing better therapeutic strategies (Colloca et al., 2017; Finnerup et al., 2021; Fitzcharles et al., 2021).

Irrespective of categorization, pathological pain negatively impacts the quality of life and remains one of the common reasons for seeking medical care (Colloca et al., 2017; Yekkirala et al., 2017; Fitzcharles et al., 2021). Despite considerable efforts to develop novel analgesics, success has been limited due to low efficacy and adverse effects (Yekkirala et al., 2017; Jayakar et al., 2021). Reasons for this include pain heterogeneity, complexity of pathophysiological mechanisms, and unreliability of preclinical models (Yekkirala et al., 2017). Although many pain states present mixed-pain characteristics, recognizing the pain type is important since neuropathic and nociplastic pain are mechanistically different from inflammatory pain and are treated differently (Colloca et al., 2017; Fitzcharles et al., 2021). Therefore, studies are needed to enhance knowledge of the mechanisms and treatment of different pathological pains.

This Research Topic aimed to compile studies addressing new insights into pathophysiological mechanisms. It also focused on pharmacological advances to treat pathological pain and reposition drugs for analgesic purposes. Four articles were successfully published (two original research articles, one clinical trial article and one review) gathering mechanisms and pharmacological targets associated with adenosine receptors, transient receptor potential melastatin (TRPM channels), toll-like receptors and potassium channels, besides the addition of dexamethasone in a multimodal analgesia approach. Such studies are briefly summarized below.

In a persistent inflammatory pain model, Cao et al. showed that microglial adenosine A2A receptor in the paraventricular thalamic nucleus regulated pain sensation and antinociceptive effects independently of opioid and cannabinoid receptors. Findings highlighted this receptor as a promising target for novel analgesic development safer and non-addictive.

In another study, using a neuropathic pain model induced by infraorbital nerve injury, Piña et al. found a role for the TRPM8 channel and voltage-gated potassium channel family 1 (Kv1) in orofacial cold allodynia. TRPM8 and Kv1, which underlies the brake current IKD (Kv1.1-1.2-dependent brake potassium current), were functionally unbalanced in trigeminal neurons after infraorbital nerve injury. This enhanced the cold sensitivity of cold thermoreceptor neurons and transformed a subpopulation of silent cold-insensitive neurons signalling pain into neurons activated by mild cold temperatures. Findings suggest that specific TRPM8 blockade and increasing the functional expression of Kv1 channels underlying IKD in trigeminal neurons could be effective tools to revert neuropathic orofacial cold allodynia.

In a double-blind randomized controlled trial (Registration: ChiCTR2100048760, www.chictr.org.cn/showproj.html?proj=130226) conducted in patients submitted to perianal surgery, Yang et al. observed that association dexamethasone with ropivacaine used in pudendal nerve block reduced moderate to severe pain during the first dressing change compared single administration of ropivacaine. It also improved sleep quality, although accompanied by prolonged numbness, without additional adverse effects. There was a rapid postoperative recovery, suggesting that this drug combination may become a novel approach to multimodal postoperative analgesia after perianal surgery.

Finally, the review by Mandlem et al. focused on TRPM2, suggesting this channel is a target for novel diagnostic and therapeutic approaches to pain management, particularly neuropathic pain. The authors discussed how TRPM2 mediates oxidative stress and lipopolysaccharide-induced Toll-like receptor 4 (TLR4)-mediated inflammation in neuronal and non-neuronal tissues and indicated that the interaction between TRPM2 and TLR4 should be considered in more studies for adequate pain management.

With the present Research Topic, we hope to provide a collection of publications that will contribute to further advance our understanding of pain neurobiology, provide opportunities to develop novel therapeutic approaches and revisit existing targets, such as ion channels and G-protein-coupled receptors (Yekkirala et al., 2017), highlighted here by TRPM2, TRPM8 and Kv1 channels and TLR-4 and adenosine A2A receptors, respectively. Together, the data included in this topic reflect the need to recognize multiple distinct specific mechanisms underlying pathological pain and identify them in each patient individually (Jayakar et al., 2021) besides the need to design new therapeutic approaches, eventually combining pre-existent treatments. While we expect that knowledge about nociplastic pain will surely increase in the future (Fitzcharles et al., 2021), this topic did not, unfortunately, receive any submission regarding this pain category, as the introduction of the descriptor “nociplastic pain” is recent (Kosek et al., 2016).
